# A Systematic Review of the Sex and Gender Reporting in COVID-19 Clinical Trials

**DOI:** 10.3390/vaccines9111322

**Published:** 2021-11-15

**Authors:** Shirin Heidari, Alice Palmer-Ross, Tracey Goodman

**Affiliations:** 1 Gender Equity and Human Rights, World Health Organisation, 1211 Geneva, Switzerland; 2 GENDRO, 1211 Geneva, Switzerland; alicepr26@gmail.com (A.P.-R.); goodmant@who.int (T.G.)

**Keywords:** COVID-19, vaccines, sex, gender, SAGER Guidelines

## Abstract

Sex and gender have implications for COVID-19 vaccine efficacy and adverse effects from the vaccine. As vaccination is one of the key responses to the COVID-19 pandemic, it is vital that sex and gender differences be acknowledged, measured, and analysed in clinical research. Here, we systematically review published COVID-19 vaccine trials, both interventional and observational, to assess the quality of reporting of sex and gender. Of the 75 clinical trials on COVID-19 vaccines included in this review, only 24% presented their main outcome data disaggregated by sex, and only 13% included any discussion of the implications of their study for women and men. Considering the sex differences in adverse events after vaccination, and the gendered aspects of vaccine hesitancy, these oversights in clinical research on vaccines have implications for recovery from the COVID-19 pandemic and for wider public health.

## 1. Introduction

In the face of the global public health emergency caused by coronavirus disease 2019 (COVID-19), a safe and effective vaccine to prevent severe disease and death and minimize further spread of the virus is crucial. Clinical research on vaccines is at the forefront of the fight against the virus. Growing research is shedding light on observed sex and gender differences in the manifestation and mortality rates of COVID-19. There is greater understanding of the biological factors underpinning the sex differences, including genetic factors and the role of estrogen in reducing mortality [1]. However, research into the different responses to COVID-19 vaccines between women and men is scarce. Past research shows there are immunological sex differences in response to self-antigens, pathogens, and vaccines [2]. For example, the seasonal trivalent inactivated influenza vaccine (TIV) has been shown to induce a stronger immune response in women than in men, with females generating “hemagglutination inhibition (HAI) antibody titres that that are twice as high as those of males” [3]. Other research indicates that women experience more frequent and severe adverse events to certain vaccines compared to men [4,5].

Aside from biological sex, socially constructed gender norms, roles, and behaviours affect exposure to the virus. Combined with other factors, such as confidence in and acceptability of vaccines, these aspects can ultimately influence vaccine effectiveness [6,7]. A more comprehensive overview of the rationale for considering sex and gender dimensions in COVID-19 vaccine research was recently published by the authors [8]. There is therefore a strong argument for sex and gender to be considered as critical variables in COVID-19 vaccine research. 

Recent studies reveal that sex and gender are poorly incorporated into COVID-19 research. A recent review of COVID-19 clinical trials showed poor reporting of sex and gender dimensions, with no justification for the lack of such analysis [9]. Many COVID-19 vaccine trials had not been published at the time of this analysis and were not captured in the previous publication. Hence, our analysis focuses specifically on COVID-19 vaccine trials, using the *Sex and Gender Equity in Research* (SAGER) *Guidelines* as an analytical framework, to assess the extent to which sex and gender dimensions have been taken into account [10]. 

## 2. Materials and Methods

We searched PubMed using the search string ((“clinical trial” [TIAB] or “trial” [TIAB] OR “study” [TIAB] OR “investigation” [TIAB] or “report” [TIAB]) AND (“vaccine” [TIAB]) AND (2019-nCoV OR 2019nCoV OR COVID-19 OR SARS-CoV-2 OR (Wuhan AND coronavirus))) to capture articles on COVID-19 vaccine clinical trials published between 1 December 2019 and 22 April 2021 [see Supplementary Materials for PRISMA Flow chart]. The inclusion criteria were: original research of interventional COVID-19 vaccine trials (Phase I, II, III, and IV), as well as published observational COVID-19 studies (e.g., post-marketing surveillance studies) and preprints in English. Interventional clinical trials were defined as trials in which unvaccinated people were administered COVID-19 vaccine candidates for the specific purpose of evaluating safety, immunogenicity, or efficacy of the vaccine. Observational trials were defined as trials where cohorts of people, including specific populations, who had already received an approved vaccine outside of the clinical trial setting were observed for effects, including immunogenicity, adverse events, safety, and effectiveness of the vaccine. Our exclusion criteria were papers that were: commentaries, reviews and articles not reporting primary data, findings from nonclinical or preclinical studies, and articles on studies with fewer than 10 participants. 

We used the SAGER Guidelines recommendations as analytical categories to examine the incorporation of sex and gender dimensions in the research and reporting of findings in peer-reviewed journals. We extracted data on the total number of participants, adult women and pregnant women, and the total number of participants and women who withdrew from the study. We also collected data on adherence to the SAGER Guidelines recommendations regarding the introduction, methodology, results (data disaggregation and sex and gender-based analysis), and discussion sections of the articles included in our systematic review. We also collected data on the trial phase, type of intervention, and funding source of the trials. 

Statistical analyses of studies by phase of trial and by funding type were performed. A previous study on HIV trials showed a differing gender balance in different phases of clinical trials [11]. We therefore grouped early (Phase I, Phase I/II Phase II) and late (Phase II/III Phase III) phase interventional trials for this analysis. We also did a grouping by size of observational trial, as observational trials did not have phase data available. Several public funding agencies, such as the National Institutes of Health (NIH), require clinical trials that are partially or fully funded by public funds to include sufficient women and men, to enable meaningful sex- and gender-based analysis. Our sample size did not permit analysis to characterize the independent associations of the three funding types. Therefore, we grouped public and mixed funding types to compare them to trials which were only privately funded. We then tested these funding type categories against gender balance and sex disaggregation of data using a one-sided Fisher’s Exact Test (significant at *p* < 0.05), as the appropriate test to assess the relationship between two variables in a small sample. While looking at the gender balance in clinical trials, we observed that some trials that included healthcare workers overrepresented women in their samples. We therefore also tested differences between women’s participation in observational trials that includedd healthcare workers vs observational trials without healthcare workers. We applied a one-tailed *t-*test, as the comparison was between two independent means (significant at *p* < 0.05). All statistical tests were performed using the Social Science Statistics website [12].

## 3. Results

In total, 1958 articles were retrieved following the PubMed search. An initial screening of titles and abstracts excluded a total of 1888 articles, including 23 articles in a language than English. The full text of the remaining 72 articles was reviewed, and we further excluded two duplicate interventional clinical trial preprints. Ultimately, 70 articles from PubMed alone met our inclusion criteria [see Supplementary Materials for PRISMA 2020 flow diagram for new systematic reviews which included searches of databases, registers and other sources]. 

The PubMed search results were then cross-referenced with data from the World Health Organization (WHO) “Draft landscape and tracker of COVID-19 candidate vaccines” for additional articles not registered in PubMed [13]. The WHO landscape tracker compiles detailed information on each COVID-19 vaccine candidate in development and monitors the progress of each clinical trial. We included an additional five interventional clinical trials which were not in PubMed. In total, we included 75 articles, of which 42 were interventional studies and 33 observational studies. 

### 3.1. General Principles of Sex and Gender Terminology

The SAGER Guidelines recommend careful use of “sex” and “gender” in order to avoid confusing the terms [14]. Sex is referred to as a biological variable, defined by X and Y chromosomes, which shape physiological mechanisms that can affect, among others, susceptibility to infection and disease, presentation of symptoms, and health outcomes. Gender relates to societal norms, roles, behaviours, and expectations of women and men, which can further shape distribution of power and resources [15]. Our analysis shows that 16% of papers use “sex” and “gender” interchangeably, with no discernible difference in their definitions. One interventional clinical trial notes six participantsto be of “non-binary sex” and does not define it is relating to gender or to sex [16]. No clinical trials considered gender aspects.

### 3.2. Recognizing the Importance of Sex and Gender in Abstracts and Introductions 

The SAGER Guidelines recommend that articles clarify in the title or abstract whether a study has been conducted with only one sex or gender. In our sample, the only single-sex study focused on pregnant and lactating women, as indicated in both the title and the abstract. The SAGER Guidelines further encourage authors to report previous studies that found sex or gender differences or similarities in the introduction [10]. While sex differences in the immune response to vaccines and pathogens are well documented, and sufficient evidence shows sex differences in risk and immune responses to severe acute respiratory syndrome coronavirus 2 (SARS-CoV-2) virus infection, only two papers (3%) mentioned sex or gender differences in their introduction. One noted how pregnant women had not been included in previous vaccine trials, and the other acknowledged previous studies which showed an association between sex and viral load [10,17,18].

### 3.3. Gender Balance in Clinical Trials

The SAGER Guidelines and, increasingly, national research funding agencies such as NIH, the Canadian Institutes of Health Research (CIHR), and the European Commission (EC), call for balanced participation of women and men in clinical trials and meaningful sex- and gender-based analysis [19,20,21]. The SAGER Guidelines further recommend that authors indicate in their methodology how they intended to recruit equal numbers of women and men in their study [10]. 

**Figure 1:**
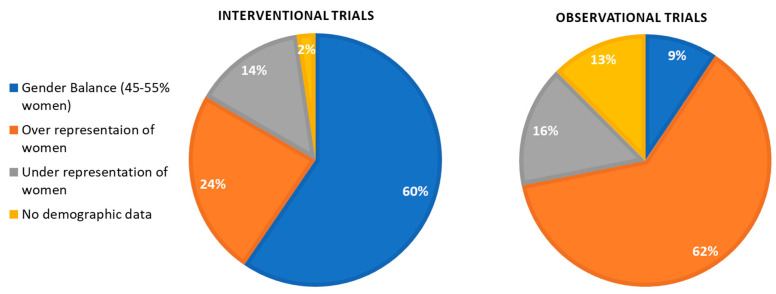
Gender balance of clinical trials by study type.

Of the 42 interventional trials, 1 did not include data on how many women and men were in the trials; and of 33 observational trials, 4 did not provide data on the number of women and men included, with 1 including only pregnant woman. These trials were therefore excluded from the analysis of gender balance in participation (Figure 1).

**Figure 2:**
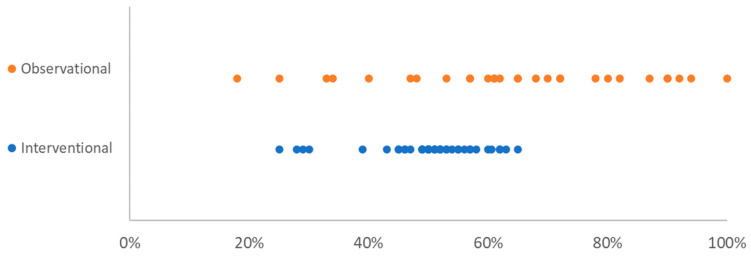
Percentage of female participation by study type. Each dot represents one study.

Of the 42 interventional trials included, 60% (25/42) were gender balanced, defined as having 45–55% female; 24% (10/41) had an overrepresentation of women (>55%); and 14% (6/41) had an underrepresentation of women (<45%) (Figure 1 and Figure 2). There were 2 interventional trials which did not provide their demographic information disaggregated by sex. In terms of providing a justification for the unequal representation of women and men in the trials, as recommended by the SAGER Guidelines, only 1 observational trial justified the predominance of women in their trials as a result of enrolling mainly healthcare workers, most of whom are women [22]. Of the 4 trials that had underrepresentation of women, which were early-phase trials (Phase I and Phase I/II) looking at safety and immunogenicity, none provided any justification for the imbalance in the trial.

There was no statistically significant difference between early (Phase I and Phase I/II) and late (Phase II/III and Phase III) interventional trials in their likelihood of being gender balanced (*p* = 0.688). Nor was there any statistically significant difference between funding source (fully or partially publicly funded vs privately funded) and the likelihood of being gender balanced (*p* = 0.453). 

Of the observational trials which noted the demographic data of their participants by sex, 71% overrepresented women in their sample (20/28), with only 11% being gender balanced (3/28) and 18% underrepresenting women (5/28) (Figure 1 and Figure 2). These figures exclude the 1 clinical trial which recruited only pregnant women. There was no statistically significant difference between large trials, defined as those greater than the median number of participants (median = 187), and small observational trials (smaller than the median) and their likelihood of having gender-balanced participation (*p* = 1.00). Only one of the studies provided justification for the higher proportion of women in their sample, explaining that in the USA, where the trial was undertaken, “nearly 75% of full-time, year-round HCWs [healthcare workers]” are women [23].

Of the five observational trials that had fewer women, three were conducted with vaccinated cohorts who were either on dialysis (*n* = 2) or had a kidney transplant (*n* = 1) and were studying humoral response. One trial assessed vaccine effectiveness for patients who suffered from chronic lymphocytic leukaemia. One of the observational clinical trials looked at the antibody response of a cohort who had had the vaccine and were exposed to new variants. None of the studies provided any justification for the underrepresentation of women. 

Interventional clinical trials were significantly (*p* = 0.00) more likely to have a gender balance in their sample than observational trials. This is likely due to 14 out of 28 (50%) of the observational trials being in healthcare settings, hence having a greater number of women participants. 

**Figure 3:**
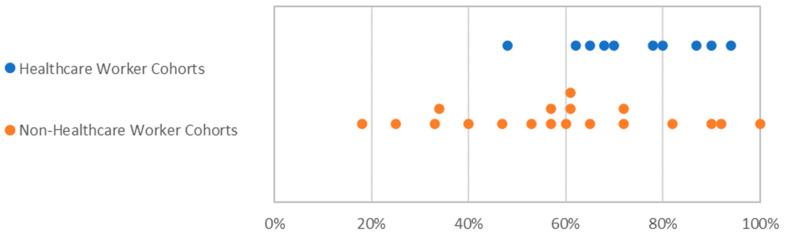
Percentage of women in observational trials with healthcare workers and non-healthcare workers. Each dot represents one study.

The difference in women’s participation in trials with healthcare worker cohorts and non-healthcare worker cohorts is shown in Figure 3. Nineteen observational trials (58%) had cohorts which did not focus specifically on healthcare workers (e.g., trials of vaccine effectiveness on patients undergoing dialysis). As expected, observational trials with healthcare workers had a statistically significant higher proportion of women (mean 74%) than those with non-healthcare workers (mean 60%) (*p* = 0.03) measured using a one-tailed *t*-test. 

### 3.4. Pregnant Women

Pregnant women were excluded from all the published interventional clinical trials in our sample, none of which justified their exclusion. One Phase III trial included nine women who had become pregnant after they received the first dose in their safety analysis. They were not offered the second dose, nor were their outcomes differentiated from the rest of the analysed population [24]. The inclusion of pregnant women in this study is only mentioned when highlighting how “[t]his report does not address the prevention of COVID-19 in other populations, such as younger adolescents, children, and pregnant women” [24].

One observational trial recruited “84 pregnant, 31 lactating, and 16 non-pregnant women” who had received the vaccine. The mean gestational age at the first vaccine dose was 23.2 weeks [17]. The study compared the vaccine-induced antibody titres between pregnant and lactating women with those of non-pregnant women to evaluate the immunogenicity of the vaccine during pregnancy and lactation. The study reported equivalent vaccine-induced antibody titres between groups and similar humoral immunity [17].

### 3.5. Trial Design 

The SAGER Guidelines recommend that “authors should report how sex and gender were taken into account in the design of the study” [10]. Of all 75 papers, only 2 (both observational) reported briefly how sex was considered in the design of the study and the analysis. One of these studies noted how they “assembled control groups of positive tests in unvaccinated patients with matching age group, sex and sampling date range” based on previous evidence that viral load could be associated with sex [25]. The second paper investigated antibody levels after vaccination in previous natural infection and infection-naïve participants, and explained in the methodology how they further assessed whether there were correlations with “gender [sic]” [18].

### 3.6. Sex-Disaggregation of Data

The SAGER Guidelines recommend that all data be disaggregated by sex, as a minimum [10]. Sex-disaggregated data should be analysed to capture potential sex or gender-based differences and to enable future meta-analysis. As previously highlighted, five studies did not specify their demographic data by sex, nor did they report their main outcome data by sex.

Out of 70 studies, only 17 (24%)–7 interventional and 10 observational trials–presented their primary outcome data by sex. There was no statistically significant difference between interventional and observational trials in their likelihood of presenting their main outcome data disaggregated by sex (*p* = 0.17), when measured using a one-sided Fisher’s Exact Test. Only 2 studies disaggregated their main outcome data by age and sex concurrently. One of these was a Phase III interventional trial of the CoronaVac vaccine, and 1 was a observational trial on patients undergoing dialysis. 

Out of the total 42 interventional trials, one paper did not disaggregate participant data by sex, so we could not tell how many women and men were involved. We therefore excluded that paper from the following analysis on sex-disaggregated data. Of the 41 interventional trials remaining, 7 disaggregated their primary outcome data by sex (17%). None of these reported statistically significant differences in vaccine efficacy, or immunogenicity, between women and men. Table 1 shows the findings of the papers that disaggregated their results by sex. While nearly all the interventional trials (40/41, 95%) reported adverse events in their samples, none presented the data by sex. While the SAGER Guidelines recommend that data on participant withdrawals also be disaggregated by sex, of 32 (78%) interventional trials that featured withdrawals, none provided information on the sex/gender of the participants who discontinued the study.

There was a statistically significant difference between early (Phase I, Phase I/II, Phase II) vs late (Phase II/III, Phase III) trials, with late phase trials being more likely to present their primary outcome data by sex (*p* = 0.017). Comparing studies fully or partially funded by public funding vs. private funding, we noted no statistically significant difference in terms of data disaggregation by sex (*p* = 0.664). 

As observational trials have less easily identifiable primary end-points, such trials were grouped by the populations they observed and the vaccine effect they were observing [31]. This included vaccine effectiveness in specific populations (*n* = 9), adverse events in previously vaccinated populations (*n* = 11), or vaccine effectiveness in vaccinated individuals (*n* = 12) [32]. Of the 29 observational trials which presented their demographic data by sex, only 34% (10/29) reported their primary outcome data by sex. Out of these 10, 5 focused on the effectiveness of the vaccine; 4 on vaccine effectiveness in populations with specific health disorders or frailties; and 1 on vaccine effectiveness in healthcare workers. The findings of these sex-disaggregated analyses are shown in Table 2.

Nearly half (48%) of observational trials reported adverse events in their populations (16/33). However, only five (31%) reported their adverse events data by sex, all of which found that women were more likely than men to suffer from adverse events. None of the observational trials in our sample included participant discontinuation. 

### 3.7. Sex- and Gender-Based Analysis 

As noted previously, only 17 out of 75 papers disaggregated their main outcome data by sex, indicating that they included sex as a variable in their analysis. This analysis mainly took the form of noting either a *p* value for statistically significant differences between women and men or a similarly narrow explanation of the observed differences. As an example, one of the papers stated only that the “sex and age of the participants did not differ their IFNγ-ELISpot [interferon gamma enzyme-linked immunospot] T-cell responses induced by vaccination” [42]. For further examples, see Table 1 and Table 2. All studies in this sample narrowly focused on biological sex differences, while gender dimensions were disregarded. There was a distinct lack of a more detailed analysis on the differences between women and men in vaccine responses and adverse effects. Sex- and gender-based analyses across age groups, vaccination arm (e.g., doses, dose intervals), and population groups (e.g., healthcare workers) were not carried out in any of the papers in our sample. 

### 3.8. Sex and Gender Implications in the Discussion Section

An important criteria of the SAGER Guidelines is that papers should discuss the implications of their study on women and men, or if a sex and gender analysis was not carried out, to address this as a limitation of their study and its generalizability [10]. Ten papers (two interventional and eight observational) (13%) in our sample adhered to the first SAGER guideline by including sex/gender as a point in their discussion.

The two interventional trials (5%) (Phase I/II trials) mentioned sex differences briefly in their discussion section. One discussed how previous studies showed sex differences in antibody titres, while the other highlighted how severe COVID-19 disproportionately affects men. 

The eight (24%) observational trials that addressed sex/gender dimensions in their discussion section included one that discussed how mainly women had received the Pfizer vaccine during their study period, justifying the overrepresentation of women in their sample; but these researchers did not discuss the implications of this overrepresentation on their findings. Two papers suggested that further research on sex differences was needed, one on pregnant women in comparison to non-pregnant women and the other suggesting further research on the impact of sex on antibody response. Two papers discussed vaccine strategies, one stating that vaccination strategies should be more inclusive of pregnant women and the other recommending that a vaccination strategy for dialysis patients should be encouraging uptake regardless of sex. The remaining three papers simply summarized their sex-related findings in their conclusions, noting that systemic reactions were associated with female sex (*n* = 1), progressive thrombotic conditions were more common in female patients (*n* = 1), and female patients with chronic lymphocytic leukaemia had better response rates to the vaccine than males (*n* = 1). 

Only 5 out of 75 papers mentioned a lack of sex- and gender-based analysis as a limitation of their study. Two interventional trials discussed how their respective studies were limited by a lack of efficacy analysis by subgroups based on sex and how the study populations lacked gender diversity. 

Three observational trials addressed the generalizability of their results. Two trials stated that results might not be generalizable because of the predominance of women in their sample. The third trial addressed a previous study which found that that 80% of adverse events were experienced by females, a finding that is potentially influenced by the fact that more women received the Moderna vaccine in that analytical period and therefore may not be generalizable.

## 4. Discussion

Our analysis shows that the majority of interventional COVID-19 vaccine studies were gender balanced. This finding indicates a remarkable improvement in women’s participation in trials compared with past studies where women have been persistently underrepresented [41,42]. In contrast, a greater proportion of observational studies of COVID-19 vaccines had an overrepresentation of women. This was due to many of the studies being conducted with healthcare workers, the majority of whom are women and who were prioritized in the early roll-out of COVID-19 vaccines. This continuing gender imbalance reflects the gendered nature of the healthcare profession, and not a gender-specific recruitment strategy of the trials. The SAGER Guidelines recommend that future clinical trials design recruitment strategies to include equal representation of women and men from the outset [8,9]. 

Sex and gender are important variables in clinical research, and are highly relevant in vaccine research. Past research has shown significant differences between women and men in susceptibility to infections, immune response to pathogens and vaccines, and frequency and severity of adverse events of vaccines [43,44,45]. A previous study on TIV, for example, reported that women given a half-dose of TIV showed a response similar to or greater than that of men given a full dose [46,47]. Several studies have also shown that women experience adverse events of vaccines to a greater extent than men [5,39]. Yet, our findings confirm recent studies which show that sex and gender implications are inadequately examined and reported in COVID-19 research. A scoping review of COVID-19 treatment trials in the first months of the pandemic by Schiffer et al. concluded that none of the studies investigated sex-specific differences in treatment efficacy [48]. Another study, by Brady et al., examining COVID-19 clinical trials, noted that of 45 published trials up to 15 December 2020, only 17.8% “report[ed] sex-disaggregated results or subgroup analyses” [49].

Our previous research, which examined the reporting of COVID-19 clinical trials on treatments and five vaccine studies, highlighted the inadequate consideration of sex and gender dimensions. Our current study, which focuses on COVID-19 vaccine trials, again shows a lack of consideration of sex and gender. While 17% of the interventional COVID-19 vaccine trials have reported overall efficacy by sex, a more detailed analysis exploring possible differences across age, ethnicity, vaccination arms, or dose intervals was not reported in any of the reviewed articles. Variables such as age and race/ethnicity can further influence susceptibility and vulnerability to infection, as well as vaccine efficacy and effectiveness [50]. However, the reporting of these additional variables is also inadequate. In our sample, only two studies disaggregated their main outcome data concurrently by age and sex. While we did not specifically extract data on the reporting of race/ethnicity, we did not note any papers that provided data by sex and race/ethnicity. 

Importantly, none of the interventional studies reported their adverse event data by sex. The observational studies in our sample that examined adverse events in vaccinated populations and reported their data by sex consistently point to greater adverse events in women. Post-marketing data, after COVID-19 vaccine roll-out, also show different experiences among women and men [5]. According to a European database that monitors adverse reactions to drugs, using data from April 2021, “88.1% of anaphylaxis cases […] after administering Pfizer-BioNTech LNP-mRNA [lipid nanoparticle packaged mRNA] COVID-19 vaccines [were] found in women” [51]. Similar data were observed after Moderna vaccinations [39]. Following AstraZeneca and Janssen vaccinations, very rare thrombosis with thrombocytopenia syndrome was also reported more frequently among women, while an increased risk of myocarditis and pericarditis has been noted among young men after Pfizer-BioNTech and Moderna vaccinations [52].

While these adverse events remain very rare, the observed sex disparities point to underlying differences between women and men that merit further investigation at the time of clinical trials. As has been reported for other vaccines, women tend to launch a stronger immune response to antigens and more frequent and severe adverse events. It may warrant investigating whether an alternative dosing strategy could induce an equivalent protective immune response in women while reducing the risk of adverse events. 

Another persistent concern is the exclusion of pregnant women from clinical vaccine research. Nearly all (99%) of clinical trials in this review excluded pregnant women, even though pregnant women have been shown to be at greater risk of severe disease and death than non-pregnant women [53]. According to a statement from the Royal College of Obstetricians and Gynaecologists, 58% of pregnant women in the United Kingdom have declined the vaccine, primarily due to safety concerns [54]. While clinical trials with pregnant women are now underway, the delay in producing sufficient clinical data on safety and efficacy of vaccines during pregnancy can have an important effect on risks and protection among this population. One in six critically ill National Health Service COVID-19 patients, who need the highest level of life-saving care, are unvaccinated pregnant women [55]. This is the population that is the most consistently excluded from clinical trials on vaccines. 

Despite increasing calls from funders, publishers, academic journals, and research governance bodies for gender-sensitive research, our results highlight how sex and gender dimensions continue to go overlooked in COVID-19 vaccine research. Several public funding agencies, such as NIH, CIHR, and the EC, encourage or require sex and gender dimensions to be meaningfully incorporated into the design, conduct, and reporting of research. However, lack of any association between studies that were fully or partially funded by public funding and sex- and gender-based analyses show that these policies remain toothless. A previous study by Curno et al. in 2016 similarly showed “[n]o significant association between the proportion of women in [HIV vaccines, treatment, and cure] trials and category of funding source” [11]. While national policies that require or even encourage gender-sensitive research are highly welcome, compliance with and enforcement of such policies need to be strengthened. A promising example is CIHR, which changed its funding policy and required all research grant applications to include a description of how sex and gender are incorporated into the design of the study, stating that this aspect will be considered in the final evaluation of proposals. Evaluation of this change shows that researchers are paying more attention to sex and gender, with clinical research showing the highest proportion of funding applications integrating sex over a 10-year period of evaluation. A further study on research quality showed that policy changes which support female scientists helped to mitigate the gendered implications of the COVID-19 pandemic. Gendered policy interventions in research, for example offering compensation for dependent caregiver costs, means that more women can undertake research. Given that women are more likely to consider sex and gender in their research, addressing inequalities in research opportunities for female scientists would help address the sex gap in medical research, both in content and authorship [56,57].

Journals themselves can play an important role in putting pressure on authors for more transparent, complete, and accurate reporting of sex and gender dimensions at the time of publication. The SAGER Guidelines offer a set of recommendations on how these dimensions should be considered and reported as a way to address the gender gap. One of the key recommendations of the SAGER Guidelines is analysis and reporting of possible sex and gender differences and, as a minimum, reporting all data by sex. Recognizing that subgroup analysis, if not appropriately designed, may not always be feasible it may reveal signals to prompt further investigations. Consistent disaggregation of all data by sex could enable meta-analyses to compare, for example, efficacy or safety of vaccines for women and men, as seen in the meta-analysis by Bignucolo et al. [58]. Yet, in our sample, rarely did any study report all data by sex, and only a quarter disaggregated data on at least one outcome by sex. Table 3 illustrates the adherence to each SAGER guideline by the studies in our sample.

We examined the quality of reporting, according to the SAGER Guidelines, in the six largest trials in our study, which account for 6.5 billion administered vaccine doses. Our analysis exposes the inadequacy of reporting sex and gender aspects in these trials (Table 4). 

Finally, most papers only considered sex as a variable and disregarded possible gender considerations. Aside from biological sex, feasibility to participate in trials, perception of risk of infection and adverse events, preference for administration route and intervals, accurate health information, and other gender-related variables can influence vaccine development and uptake [61,62]. Vaccine hesitancy and resistance are threats to public health, which is why it is imperative that both sex and gender be considered as key variables in studies on vaccine safety, efficacy, and adverse effects, and in studies on acceptability and uptake. 

Increasingly, reports are emerging that there are sex and gendered effects from the vaccine that are not being reported in the original clinical trials. A recent example is the effect of the COVID-19 vaccine on menstruation, with over 30,000 reports of changes to periods and unexpected vaginal bleeding [63]. Developments like this strengthen the argument that data should be routinely collected in clinical trials on menstruation changes, which is currently not the case. Due to sex- and gender-blind data gathering and reporting practices, important events like menstrual changes are not considered in clinical research. 

## 5. Conclusions

Our paper has some limitations. First, our analysis included articles that were published up to 22 April 2021, and only included articles published in English. Second, we only included articles that were published in PubMed or were available in the WHO “Draft landscape and tracker of COVID-19 candidate vaccines”. While we are confident that this approach was sufficient to capture the majority of the published and preprinted findings of COVID-19 vaccine trials, some articles may not have been identified. 

Overall, our findings underline the inadequate reporting of sex and gender dimensions in COVID-19 clinical research. While support for the SAGER Guidelines has been steadily growing, and many prominent journals and publishers have committed to upholding the key principles, our findings suggest more work needs to be done to reinforce such policies and to ensure that sex and gender dimensions are adequately and consistently considered in research and reported as a standard requirement across journals. Capacity building of peer reviewers and incorporation of the guidelines in the evaluation process can be one powerful approach to improve reporting. 

Other recommended actions included in the recent publication in *BMJ Global Health* serve as a timely reminder about the importance of considering sex and gender and their intersection with other important variables early on, from research design to vaccine delivery [8]. Curbing the pandemic requires an effective approach to maximize confidence in vaccines for all and minimize any barriers to vaccination. More systematic adherence to the SAGER Guidelines is an important step in encouraging more complete and transparent reporting of sex and gender dimensions, mainstreaming of these aspects in design and conduct, and better science that serves all.

## Figures and Tables

**Table 1:** Description of articles on interventional clinical trials that provided data on at least one outcomes by sex.

Citation	Phase	Primary End-Points *	Outcome Data by Sex **	Vaccine	Quotes
Ewer et al., 2021 [26]	Phase I/II	Efficacy and safety	Immune response	ChAdOx1 nCoV-19 (AZD1222)	“We found no sex difference in vaccine response at any of the time points measured”
Zhu et al., 2021 [27]	Phase II	Adverse reactions and antibody response	Antibody response	Adenovirus type-5 (Ad5)-vectored	“Sex and age of the participants did not differentiate their IFNγ-ELISpot T-cell responses induced by vaccination”
Logunov et al., 2021 [28]	Phase III	Efficacy	Immunogenicity	rAd26 and rAd5 vector-based heterologous prime-boost	“Antibody levels did not differ significantly between men (*n* = 179) and women (*n* = 159; *p* = 0.258).”
Baden et al., 2021 [29]	Phase III	Efficacy and safety	Efficacy	mRNA-1273	“The vaccine efficacy to prevent COVID-19 was consistent across subgroups stratified by demographic and baseline characteristics: age groups (18 to <65 years of age and ≥65 years), presence of risk for severe COVID-19, sex […]”
Polack et al., 2020 [24]	Phase III	Efficacy and adverse events	Efficacy	BNT162b2 mRNA	“Similar vaccine efficacy (generally 90–100%) was observed across subgroups defined by age, sex, race, ethnicity, […]”
Ella et al., 2021 [30]	Phase I/II	Efficacy and adverse events	Efficacy	BBV152	“Seroconversion rates and GMTs across three age groups (≥12 to <18 years, ≥18 to <55 years, and ≥55 to ≤65 years) and between both sexes were similar, but only small numbers of participants were included in the youngest and oldest age groups“
Sadoff et al., 2021 [16]	Phase III	Efficacy	Efficacy	Ad26.COV2.S	“No meaningful differences in vaccine efficacy were observed among subgroups defined according to sex, race, or ethnicity.”

COVID-19: coronavirus disease 2019; GMT: geometric mean titre; IFNγ-ELISpot: interferon gamma enzyme-linked immunospot; mRNA; messenger RNA. * “Primary end-points” are taken from the clinical trials registration information. ** The category “outcome data disaggregated by sex” is based on the type of data researchers have disaggregated. So even if a trial was looking at efficacy and safety, researchers may only ever have reported their efficacy data disaggregated by sex and not their safety data too.

**Table 2:** Description of articles reporting observational clinical trials that provided data on at least one outcome by sex.

Citation	Objective	Primary Outcome	Quotes	Statistically Significant Difference?
McMahon et al., 2021 [33]	Cutaneous reactions to vaccine	Adverse events	“Ninety per cent of the vaccine reactions were reported in female patients.”	Yes
Greinacher et al., 2021 [34]	Thrombotic thrombocytopenia after vaccination	Adverse events	“Among these patients, the median age was 36 years (range, 22–49); 9 of 11 were women. All the patients presented with concomitant thrombocytopenia (median nadir of platelet count, approximately 20,000 per cubic millimetre; range, 9000–107,000).”	Yes
Salmerón Ríos et al., 2021 [35]	Vaccine efficacy in frail or disabled nursing home residents	Effectiveness	“Frailty, disability, older age, sex, cognitive impairment, and comorbidities were not associated with different antibody titres.”	No
Boyarsky et al., 2021 [36]	Immunogenicity in solid organ transplant patients	Effectiveness	“The immune response to the vaccine by sex was found to have a *p* value = 0.60 and therefore sex is not statistically significant.”	No
Shimabukuro, 2021 [37]	Allergic reactions after the Moderna vaccine (December–January)	Adverse events	“The clinical and epidemiological characteristics of anaphylaxis case reports after receipt of the Moderna COVID-19 vaccine are similar to those reported after receipt of the Pfizer-BioNTech COVID-19 vaccine (5). […] A strong female predominance of anaphylaxis case reports exists for both vaccines.”	Yes
Lacson et al., 2021 [38]	Immunogenicity in patients undergoing dialysis	Effectiveness	“Factors associated with poor seroconversion in our cohort include female sex, younger vintage, potential immunosuppression from diseases, transplant, or medications, [Congestive Heart Failure], and covaccination and hospitalization during the peri-vaccination period.”	Yes
Shimabukuro, 2021 [39]	Allergic reactions after the Pfizer-BioNTech vaccine (December only)	Adverse events	“A strong female predominance of anaphylaxis case reports exists for both vaccines.”	Yes
Ou et al., 2021 [40]	Immunogenicity in solid organ transplant patients	Adverse events	“Females were more likely to experience systemic symptoms after either dose.”	Yes
Street et al., 2021 [41]	Efficacy in patients with chronic lymphocytic leukaemia	Effectiveness	“In a univariate analysis (this table), the variables found to be significantly associated with response included: younger age (≤65 years), female sex, early disease stage (Binet stage A), mutated IGHV, b2-microglobulin (≤3.5 mg/L), untreated/off-therapy ≤ 12 months from the last anti-CD20 therapy, IgG levels ≤ 550 mg/dL, IgM levels ≤ 40 mg/dL, and IgA levels ≤ 80 mg/dL.”	Yes
Padoan et al., 2021 [25]	Antibody response in a cohort of characterized healthcare workers	Effectiveness	“No significant anti-S-RBD level differences were found between males and females in any of the studied conditions.”	No

CD20: B-lymphocyte antigen CD20; IGHV: immunoglobulin heavy chain variable region genes; IgA: immunoglobulin A; IgG: immunoglobulin G; IgM: immunoglobulin M; S-RBD: anti-spike protein receptor-binding domain.

**Table 3:** Adherence to SAGER Guidelines.

Scheme	No. of Interventional Vaccine Trials Complying with the Recommendation	No. of Observational Vaccine Trials Complying with the Recommendation	Overall %
1. Introduction: Sex and gender differences in the infection, manifestation, or outcomes of COVID-19 should be acknowledged in the introduction.	0/42	2/33	3%
(2a) Methodology: Papers should report how sex and gender were taken into account in the design of the study.	0/42	2/33	3%
(2b) Methodology: Papers should justify reasons for the exclusion, or differing numbers, of males or females.	1/42	1/33	3%
(3a) Results: “Sex- and gender-based analyses should be reported regardless of positive or negative outcome” [10]. Articles should note if there is a difference between sexes or genders, or if there is no difference, in their results.	7/41	10/29	24%
(3b) Results: Articles should report all their data disaggregated by sex.	7/41	10/29	24%
(4) Discussion: What the results of the study mean for women and men should be analysed in the discussion section.	2/42	8/33	13%
(5) Generalizability: If a sex and gender analysis is not done, this should be justified or addressed in relation to the generalizability of the results.	2/42	3/33	7%

**Table 4:** Adherence to the SAGER Guidelines by the six main Phase II/III and Phase III trials in our sample.

Name of Vaccine (Ref.)	Phase	Introduction	Methodology	Sex Disaggregation	Discussion	Generalizability	No. of Doses *
CoronaVac [59]	III	No	No	No	No	No	367 million
AstraZeneca ** [60]	II/III	No	No	No	No	No	3009 million
Sputnik V [28]	III	No	No	Yes (immunogenicity)	No	No	765 million
Moderna [29]	III	No	No	Yes (efficacy)	No	No	816 million
Pfizer-BioNTech [24]	III	No	No	Yes (efficacy)	No	Yes	1220 million
Janssen [16]	III	No	No	Yes (efficacy)	No	No	368 million

** Number of doses ordered as of 28 May 2021 based on data from Statistica (59); * Phase III AstraZeneca paper yet to be published as of 22 April 2021 when our search was conducted.

## Data Availability

No new data were created in this study. The PRISMA Flow chart and the full search string used are available in Supplemental Material Figure S1.

## Supplementary Materials

The following are available online at https://www.mdpi.com/article/10.3390/vaccines9111322/s1, Figure S1: title: PRISMA 2020 flow diagram for new systematic reviews which included searches of databases, registers and other sources.
