# Supplementary material for: A Systematic Review of the Sex and Gender Reporting in COVID-19 Clinical Trials
Source: Vaccines (Basel). 2021 Nov 15;9(11):1322. doi: 10.3390/vaccines9111322 (PMC8622702; doi:10.3390/vaccines9111322)
Supplement: Supplementary file 1 [file vaccines-09-01322-s001.zip › vaccines-1418164-supplementary.pdf]

PRISMA 2020 flow diagram for new systematic reviews which included searches of databases, registers and other sources

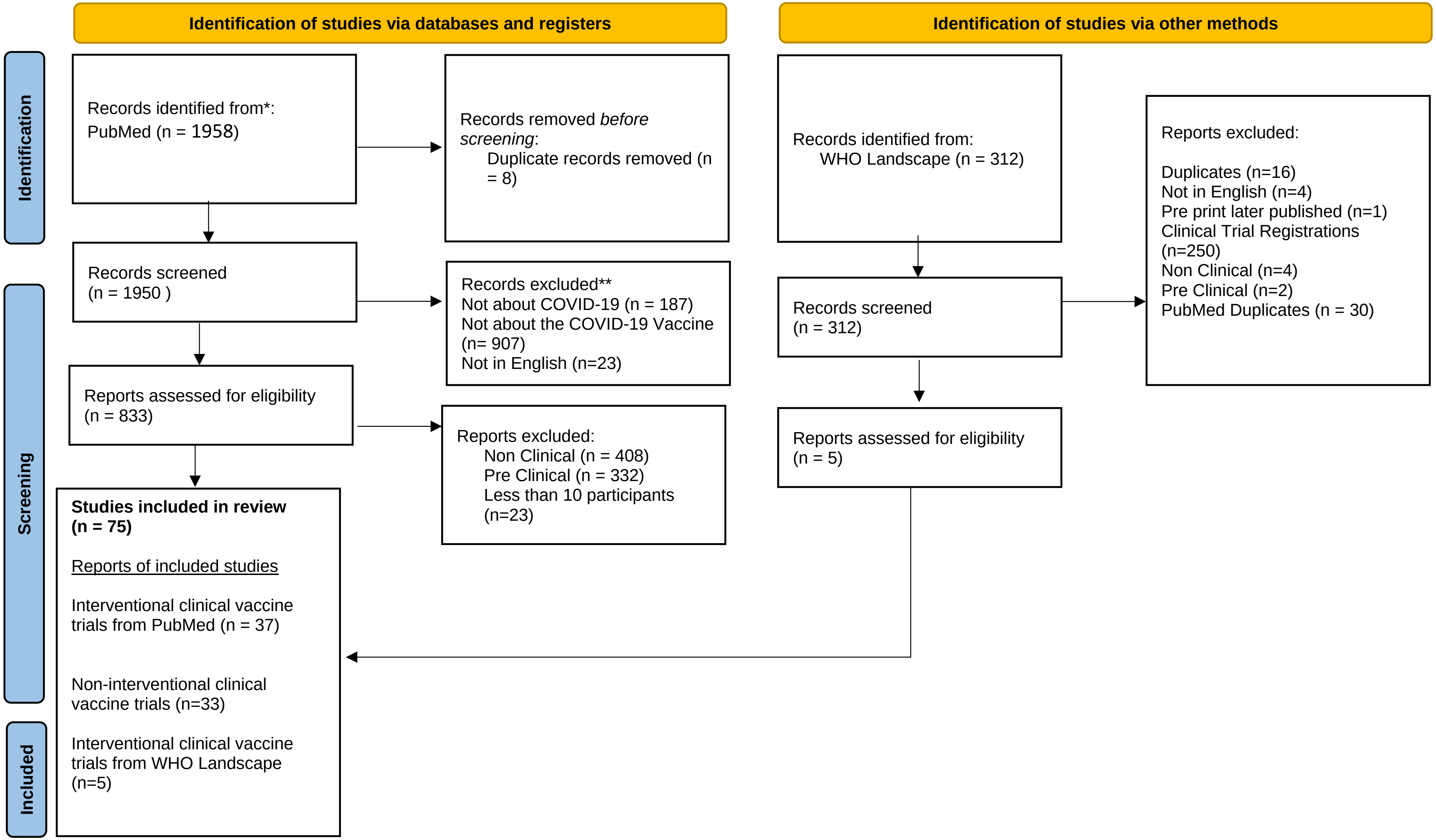

From: Page MJ, McKenzie JE, Bossuyt PM, Boutron I, Hoffmann TC, Mulrow CD, et al. The PRISMA 2020 statement: an updated guideline for reporting systematic reviews. BMJ 2021;372:n71. doi: 10.1136/bmj.n71. For more information, visit: http://www.prisma-statement.org/
